# An Overview of the Management of Drug-Resistant Tuberculosis in Six French-Speaking African Countries from 2018 to 2022

**DOI:** 10.3390/tropicalmed9080176

**Published:** 2024-08-09

**Authors:** Gisèle Badoum, Abdoul R. Ouédraogo, Attannon A. Fiogbé, Albert Kuate Kuate, Alphazazi Soumana, Yacine Mar Diop, Mohammed F. Dogo, Olivia B. Mbitikon, Adjima Combary, Gildas Agodokpessi, Dissou Affolabi, Annie Bisso, Dona R. Atimbada, Sonia Menon, Kobto G. Koura

**Affiliations:** 1 Service de Pneumologie, Centre Hospitalier et Universitaire de Yalgado Ouédraogo (CHU-YO), Ouagadougou P.O. Box 7022, Burkina Faso; 2 Unité de Formation et de Recherche en Sciences de la Santé, Université Joseph Ki-Zerbo, Ouagadougou P.O. Box 7021, Burkina Faso; aouedraogo.consultant@theunion.org; 3 International Union Against Tuberculosis and Lung Disease, 75001 Paris, France; arnauld.fiogbe.consultant@theunion.org (A.A.F.); albert.kuatekuate.consultant@theunion.org (A.K.K.); alphazazi.soumana.consultant@theunion.org (A.S.); yacine.diop.consultant@theunion.org (Y.M.D.); fall.dogo.consultant@theunion.org (M.F.D.); olivia.mbitikon.consultant@theunion.org (O.B.M.); dona.atimbada.consultant@theunion.org (D.R.A.); sonia.menon.consultant@theunion.org (S.M.); 4 Service de Pneumologie, Centre Hospitalier et Universitaire de Tengandogo (CHU-T), Ouagadougou P.O. Box 104, Burkina Faso; 5 National Tuberculosis Programme, Cotonou P.O. Box 321, Benin; aggildas2@gmail.com (G.A.); affolabi_dissou@yahoo.fr (D.A.); 6 Service de Pneumologie, Centre National et Universitaire de Pneumo-Phitsiologie de Cotonou (CNHUPP/C), Cotonou P.O. Box 321, Benin; 7 National Tuberculosis Programme, Yaoundé P.O. Box 15656, Cameroon; anniebisso@pnlt.cm; 8 National Tuberculosis Programme, Niamey P.O. Box 623, Niger; 9 National Tuberculosis Programme, Dakar P.O. Box 4024, Senegal; 10 National Tuberculosis Programme, Lomé P.O. Box 2171, Togo; 11 National Tuberculosis Programme, Ouagadougou P.O. Box 6632, Burkina Faso; adjimacombary859@gmail.com; 12 Epitech Research, 1160 Auderghem, Belgium; 13 UMR261 MERIT, Université Paris Cité, IRD, 75006 Paris, France

**Keywords:** drug-resistant tuberculosis, treatment outcomes, French-speaking African countries

## Abstract

Drug-resistant tuberculosis (DR-TB) poses a significant public health challenge, particularly in resource-limited settings. The prevalence and management of DR-TB in African countries require comprehensive strategies to improve patient outcomes and control the spread of the disease. Aggregated routine data (from 2018 to 2022) on multidrug-resistant TB (MDR-TB) were collected from the National TB Programs (NTPs) from all six countries. The diagnostic capacity for MDR-TB was globally insufficient. The system for collecting and transporting samples was sometimes inoperative. A total of 2353 cases of MDR-TB were reported, with 86.4% receiving treatment. The gap between the expected number of MDR-TB cases and the number reported per country varied from 51.5% to 88.0%, depending on the year. Fifty-two extensively drug-resistant (XDR) TB cases received treatment regimens over five years, with variations across countries. All patients received free follow-up examinations, nutritional and financial support for travel expenses to the outpatient care and treatment centers. The MDR-TB treatment success rates for all regimens between 2018 and 2021 ranged from 44.4 to 90.9%, varying by country and year. The information system relied on primary tools, reporting tools, and digital solutions. Progress has been made in MDR-TB management; however, challenges persist, necessitating resources to enhance access to rapid molecular screening tests.

## 1. Introduction

Tuberculosis (TB) remains a global health challenge, exacerbated by the emergence of drug-resistant strains. Drug-resistant TB (DR-TB) is a person with TB disease who is infected with a strain of *Mycobacterium tuberculosis* complex that is resistant to any TB medicines tested [1]. DR-TB includes (1) Isoniazid-resistant and rifampicin-susceptible TB (Hr-TB; the strain of *M. tuberculosis* complex is resistant to isoniazid but susceptible to rifampicin); (2) Rifampicin-resistant TB (RR-TB; the strain of *M. tuberculosis* complex is resistant to rifampicin); (3) Multidrug-resistant TB (MDR-TB; the strain of *M. tuberculosis complex* is resistant to both rifampicin and isoniazid); (4) Pre-extensively drug-resistant TB [pre-XDR-TB; the strain of *M. tuberculosis complex* is resistant to rifampicin (and which may also be resistant to isoniazid), and which is also resistant to at least one fluoroquinolone (either levofloxacin or moxifloxacin)]; (5) Extensively drug-resistant TB [XDR-TB; the strain of *M. tuberculosis* complex is resistant to rifampicin (and which may also be resistant to isoniazid), as well as resistant to at least one fluoroquinolone (levofloxacin or moxifloxacin) and at least one other “Group A” drug (bedaquiline or linezolid)] [1]. MDR-TB is a subset of RR-TB, and the two are often grouped together using the term MDR/RR-TB [1]

In 2022, the World Health Organization (WHO) estimated that there would be around 410,000 new cases of multidrug-resistant tuberculosis (MDR-TB) and rifampin-resistant TB (RR-TB) worldwide (95% uncertainty interval [UI]: 370,000–450,000) [2]. In the same year, 22,495 cases were reported in the African region, 97% of which were under treatment [2]. The 2020 cohort of MDR-TB patients in the African region showed that 71% were successfully treated [2]. Despite the funding mobilized for TB, improved drug availabilitý and quality, technical assistance, and capacity building, drug-resistant TB (DR-TB) remains a threat to public health worldwide.

The reasons for the persistence of TB in the world, including Africa, encompass poverty, demographic growth, and human migration, as well as HIV infection, which is particularly prevalent in impoverished nations, where 95% of TB cases are concentrated [2]. The true burden of MDR-TB remains poorly understood in certain sub-Saharan countries due to the lack of routine surveillance, inter-country coordination, and poor reporting. In response to this situation, the WHO has put in place strategies to combat TB and MDR-TB in particular [2]. Against this backdrop, the International Union Against TB and Lung Disease (The Union) has been implementing the Contributing to the Elimination of TB in Africa (CETA) project since 2019 in eight French-speaking African countries. One of the components aims to enhance care provision, particularly for patients suffering from MDR-TB. Conducted within the framework of the CETA project, this study offers an overview of the DR-TB management landscape and an in-depth analysis of its strengths and weaknesses, aimed at enhancing program management in French-speaking Africa.

## 2. Materials and Methods

### 2.1. Study Design, Study Site, and Study Population

This is a cross-sectional analysis of DR-TB surveillance data from 1 January 2018 to 31 December 2022.

This study was carried out in six of the eight French-speaking African countries in the CETA project (Benin, Burkina Faso (BF), Cameroon, Niger, Senegal, Togo). All states, except Cameroon, are included in the list of Priority Poor Countries that France is prioritizing as part of its solidarity efforts [3]. The selection of these countries is determined by a thorough assessment of their needs, their willingness to engage in the project, the EU’s familiarity with them, alongside their capacity to effectively manage financing from the Global Fund.

### 2.2. Data Collection

Using a standardized file, we collected and compiled routine data on DR-TB from the monitoring and evaluation departments of the National TB Programs (NTPs) of the countries participating in this study.

This study looked at key indicators of DR- TB, including case notification, treatment initiation rates, and treatment outcomes, along with the inherent strengths and weaknesses in managing this disease within each country. All countries adhere to the WHO operational definitions, which have been incorporated into their national guidelines for managing MDR-TB [4].

### 2.3. Data Analysis

Aggregate data were collected using standardized forms designed for the CETA project. The data were compiled by country and by year using Microsoft Excel 2016 for Windows. Statistical analyses and tables were produced using Microsoft Excel 2016 for Windows.

### 2.4. Ethics

This study was conducted under programmatic conditions, relying on aggregated instead of personal data. Formal approval from ethics committees of the respective countries was not necessary for this purpose. The data provided by NTPs were used with the consent of program managers.

## 3. Results

### 3.1. Screening Strategy

#### 3.1.1. Diagnostic Tests and Indications

Screening strategies depend on the target population and the countries’ diagnostic capacities in terms of the availability of diagnostic tests for MDR-TB (Table 1 and Table 2).

The indications for Xpert MTB/RIF tests in the six countries were as follows: (1) presumed DR-TB patients (contact subjects of DR-TB; patients treated after having been lost to follow-up; relapse cases; positive controls at M2, M3, M5, and M6; (2) presumed TB patients from populations at high risk of TB (people living with HIV; children; prisoners; gold miners; people aged 65 and over; individuals with diabetes; people with kidney failure; health workers); (3) new smear-positive patients; and (4) any patient suspected of having TB (laboratory with Xpert MTB/RIF test).

Xpert MTB/RIF test deployment as the primary screening method for all presumptive TB patients started in Benin in 2018, followed by BF in 2020, Togo and Senegal in 2021, and Cameroon and Niger in 2022.

#### 3.1.2. Sample Collection and Transport System

Depending on the country, samples were transported either by health workers or NGOs, supported financially by partners, notably the Global Fund. Alternatively, an integrated system facilitated the transportation of biological samples from district laboratories to those equipped with a GeneXpert MTB/RIF device, using postal services or private carriers. Sample referral in the six countries was organized as follows: (1) from health facilities lacking a laboratory to CDTs with a bacilloscopy laboratory; (2) from laboratories conducting bacilloscopy to GeneXpert sites; and (3) from GeneXpert sites to the National Reference Laboratory (NRL).

#### 3.1.3. Drug-Resistant TB Notification

##### Number of Confirmed MDR-TB Cases Treated from 2018 to 2022 in the Six Countries

Over the span of five years, a total of 2353 cases of MDR-TB were reported in the six countries, with 2033 patients (86.4%) receiving treatment. The number of confirmed MDR-TB cases ranged from 72 in Togo to 854 in Cameroon. The proportion of treated MDR-TB cases ranged from 77.4% in Togo to 91.6% in Senegal (Table 3).

The gap between the expected number of MDR-TB cases and the number of cases reported per country ranged from 51.5% to 88.0%, depending on the year (Table 4).

##### Number of Confirmed XDR-TB Cases Put on Treatment from 2018 to 2022

In the past five years, all 57 confirmed XDR-TB cases have been put on treatment. The number of confirmed XDR-TB cases ranged from one 1 case in Benin to 17 cases in Niger (Table 5).

### 3.2. Treatment Strategy

#### 3.2.1. Treatment Protocols

Standardized treatment is routinely applied to all patients across countries. The treatment regimens differ for MDR-TB and XDR-TB. All countries have switched to a short, all-oral regimen (Table 6).

#### 3.2.2. Organization of Care

The number of treatment centers for MDR-TB varied from one center (in Benin and Togo) to eleven centers in Cameroon. Across all countries, there was a predominant shift towards decentralization and outpatient follow-up, except for Benin, where strict hospitalization was enforced throughout the treatment period. Additionally, in all countries, patients benefited from complementary follow-up examinations and received nutritional and financial support to cover travel expenses related to outpatient care (Table 7).

#### 3.2.3. MDR-TB and Extensively DR-TB (XDR-TB) Treatment Results

The overall therapeutic success was 71.5% in 2018 and decreased to 62.7% in 2020. Togo and Benin were the only countries which witnessed an increase in therapeutic success, 81.8% and 90.9%, respectively (Table 8 and Table 9).

### 3.3. Information System

The information system is based on the country’s health pyramid (central level, intermediary level, and peripherical level). The information system is based on primary tools, reporting tools, and paperless solutions. All the NTPs have a data management department, which is closely linked to the intermediary and peripheral level (Table 10). Aggregated data are collected quarterly at TB clinics and then sent to the regional level, where checking is carried out before transmission to the central level.

## 4. Discussion

The emergence of resistance to anti-TB drugs is a global threat to TB control efforts. This descriptive study shows a gap of 77% to 79.1% between the reported number of MDR-TB cases and the estimated number of cases in the six countries from 2018 to 2021, falling below the WHO’s estimated incidence rates [2,4,5,6,7,8]. Notably, a consistent disparity persists between WHO estimates and the number of MDR-TB cases detected [2,4,5,6,7,8]. 

Challenges in accessing services, including geographical and financial barriers, poverty, and limited education, along with patient attrition between services (laboratory, clinical, and follow-up), appeared to contribute to the under-reporting of cases [9,10]. The CETA project aims to share best practice and innovative approaches for enhancing MDR-TB notification. One component involved active case finding among presumed TB patients at all entry points to TB Diagnostic and Treatment Centers (DTCs) within the community, offering the potential to bridge current gaps in MDR-TB case detection and reporting in these six countries, which were likely impacted by the COVID-19 pandemic. The decrease in MDR-TB case detection and reporting from 2019 to 2020 reflects disruptions to TB diagnosis and treatment services caused by the pandemic, affecting healthcare delivery on both the supply and demand sides. According to the WHO, in 2020, only 71% (2.1/3.0 million) of people diagnosed with pulmonary TB worldwide were tested for rifampicin resistance. This led to the diagnosis of 132,222 cases of MDR or RR-TB and 25,681 cases of pre-XDR TB (resistance to rifampin, isoniazid and fluoroquinolones, or Pre-XDR-TB), amounting to 157,903 cases. This is a sharp decrease (22%) from the 201,997 people detected with DR-TB in 2019 [8].

Enhancing the notification rate of MDR-TB hinges on promptly diagnosing cases. Following WHO guidelines, diagnosing MDR or RR-TB requires both the bacteriological confirmation of TB and the identification of DR using rapid molecular tests or culture methods [11]. The WHO recommends that all individuals with signs and symptoms of TB should first receive a rapid molecular diagnostic test and specifically Xpert MTB/RIF Ultra and Truenat [11], given their superior accuracy in detecting both simple and DR-TB. 

All six countries in our study had access to Xpert tests (Xpert MTB/RIF, Xpert MTB/RIF Ultra, Xpert MTB XDR); Hain tests (LPA1 or GenoType MTBDR plus 2.0, LPA2 or GenoType MTBDR plus); and culture with phenotypic drug susceptibility testing. The use of the Xpert MTB/RIF test as a first-line screening test for all presumed TB patients in laboratories with Xpert MTB/RIF tests began in 2018 in Benin, in 2020 in Burkina Faso, in 2021 in Togo, and in 2022 in Cameroon and Niger. However, there has been a shortage of GeneXpert equipment in all countries. In Cameroon, only 36 out of 306 laboratories have access to GeneXpert equipment, which has hindered the diagnosis of MDR-TB, consequently leading to low case reporting. Additionally, another hindrance to diagnosis was the underdeveloped state of sample transport systems in most countries, which require closer assessment to determine their effectiveness. Potential differences in identification and treatment between countries and across years may be attributed to disparity in the availability of diagnostic tools, healthcare workers, distribution and capacity of healthcare facilities, and challenges posed by rural versus urban settings. Furthermore, there was no harmonized protocol across the countries.

Indeed, there is a centralized system in place for transporting samples from peripheral health facilities lacking laboratories to DTCs equipped with bacilloscopy laboratories, then from these laboratories to GeneXpert sites, and finally from GeneXpert sites to the National Reference Laboratory for Mycobacteria. However, this sample transport system still faces logistical challenges, especially concerning the time required for sample transportation and the timely delivery of results. The implementation of Xpert platform interconnection software, particularly DataToCare, aims to link national laboratory networks by gathering diagnostic and patient data at the TB center level. This software displays information in real-time on a dashboard, aiding central-level decision-making, and promptly reporting test results to medical teams and patients. While applications are available, they do not consistently operate optimally in BF, Cameroon, Niger, and Togo.

The proportion of MDR-TB cases among those detected in our study who received treatment varied from 77.4% in Togo to 91.6% in Senegal. However, there was a huge gap between the expected number of MDR-TB cases and the number of cases reported by country, which varied from 51.5% to 88.0% depending on the year. Worldwide, only around a third of people with DR-TB had access to treatment by 2021. The cumulative total number of people with MDR/XDR-TB worldwide, who were actually receiving treatment in 2018–2020 was 482,683, or only 32% of the five-year target (2018–2022) of 1.5 million [8]. For children, the cumulative number was 12,219, or only 11% of the five-year target of 115,000 [8]. In 2020, 150,359 individuals diagnosed with MDR/XDR-TB were put on treatment, marking a 15% decrease from the total of 177,100 in 2019. This decline may be attributed to the impact of the COVID-19 pandemic [8]. The main reasons for not starting treatment were refusal of care and death, and thus, the need for awareness-raising initiatives to combat stigma to improve treatment uptake is evident. 

Standardized treatment is routinely applied to all patients across countries. All countries have switched to a short, all-oral regimen in line with WHO recommendations [12]. In 2022, new WHO guidelines recommended a six-month BPaLM/BPaL treatment regimen for eligible patients [12]. The shorter duration, reduced number of doses, and high efficacy of this new treatment regimen have the potential to alleviate the burden on healthcare systems and conserve valuable resources. As a corollary, extending diagnostic and therapeutic coverage to all individuals in need would become feasible, whereas previously, treating a patient with MDR-TB required nine to twenty months [13]. However, a potential challenge in implementing this recommendation would be their capacity to conduct the bedaquiline (Bdq) sensitivity test before initiating the new short oral regimen. The emergence of strains that are resistant to Bdq is a notable concern, with a recent study conducted in Moldova reporting a 15% prevalence of resistance to Bdq within their MDR-TB cohort [14].

The number of MDR-TB treatment centers varies from one country to another, with one center in Benin and Togo to eleven centers in Cameroon. Recognizing the reluctance of some patients to visit treatment centers, often due to prejudice or limited resources, there is an emerging trend in all countries towards decentralization and outpatient treatment.

However, this decentralization effort faces challenges in effectively supporting patients to ensure consistent TB care, potentially fostering the emergence of new forms of resistance. Another obstacle lies in the insufficient identification and management of adverse effects caused by anti-TB drugs, resulting in treatment interruptions and attrition. In Cameroon, patients are required to pay upfront for drugs to manage adverse effects. Nevertheless, despite these challenges, patients in all countries receive free follow-up examinations and access to nutritional and financial support. 

Furthermore, the under-reporting of adverse drug reactions to national pharmacovigilance committees is a prevalent issue across countries. Consequently, with the implementation of decentralization, it is expected that most countries experience a potential decline in key indicators related to MDR-TB management, and thus, the implementation a well-conceived decentralization model may require increased resource allocation.

To effectively combat TB, key indicators of success include ongoing research, treatment of individuals with TB, including MDR-TB, improved accessibility to high-quality DTCs, and intensified prevention initiatives [15]. The global targets are treatment coverage for MDR-TB and a treatment success rate of 90% by 2025 [15]. In our study, treatment success rates exhibited variability both between countries and across years, ranging from 44.4% in Cameroon in 2020 and Togo in 2021 to 86.2% in Benin in 2019. Worldwide, the treatment success rate for patients who were initiated on MDR-TB treatment displayed an upward trend, increasing from 48% in 2009 to 58% for the 2017 cohort [6].

Our study warrants careful consideration of both its strengths and limitations. By examining multiple countries over five years, we were able to provide an overview of regional trends, challenges, and progress in MDR-TB diagnosis and treatment. This approach allowed us to identify cross-country patterns and potential regional strategies for improvement. Furthermore, analyzing data over several years helps us understand the impact of interventions and policy changes, generating hypotheses for future sub-analyses. 

However, apart from these abovementioned strengths, a key limitation is that the study is programmatic and descriptive, serving as preliminary research rather than an in-depth analysis. Thus, only aggregate data were collected, precluding us from assessing which socio-economic and clinical factors were associated with MDR-TB. This limitation underscores the need for further detailed studies to explore the underlying mechanisms driving spatial variations, which could help tailor strategies to address specific local constraints. Additionally, future research should collect data on HIV patients and other vulnerable populations to better understand their impact on MDR TB and XDR TB outcomes.

## 5. Conclusions

Considerable progress has been made in the management of MDR-TB in the various CETA project countries. Nevertheless, MDR-TB remains a challenge in terms of screening and management, which requires significant technical and financial resources to improve access to rapid molecular screening tests. Additional support holds promise for improving existing systems and outcomes. Emphasizing knowledge exchange and skill pooling between countries through collaborative frameworks is essential. Despite the recommendation of new treatment regimens such as BPaL/BPaLM in 2023, their adoption has yet to materialize in the region.

## Figures and Tables

**Table 1:** Availability of diagnostic tests for MDR-TB by country, December 2022.

Country	Available Diagnostic Tests for MDR-TB					
	Xpert MTB/RIF	Xpert MTB/RIF ULTRA	Line Probe Assay for First-Line Drugs (FL-LPA)	Line Probe Assay for Second-Line Drugs (SL-LPA)	Loop-Mediated Isothermal Amplification (TB-Lamp)	Culture and Sensitivity Tests
Benin	X	X	X	X	X	X
Burkina Faso	X	X	X	X		X
Cameroon	X	X	X	X	X	X
Niger	X	X	X	X		X
Senegal	X	X	X	X		X
Togo	X	X	X	X		X

**Table 2:** Distribution of the number of microscopy units and GeneXpert by country from 2018 to 2021.

Country		Year			
		2018	2019	2020	2021
Benin	Number of GeneXpert	07	29	30	30
	Number of microscopy units	79	84	89	92
Burkina Faso	Number of GeneXpert	15	15	18	37
	Number of microscopy units	111	111	120	128
Cameroon	Number of GeneXpert	24	32	33	36
	Number of microscopy units	255	261	265	306
Niger	Number of GeneXpert	13	21	31	49
	Number of microscopy units	221	235	238	262
Senegal	Number of GeneXpert	22	24	48	53
	Number of microscopy units	132	142	152	153
Togo	Number of GeneXpert	15	15	15	43
	Number of microscopy units	69	69	82	89
All countries	Number of GeneXpert	96	136	175	248
	Number of microscopy units	867	902	946	1030

**Table 3:** Number of confirmed MDR-TB cases put on treatment from 2018 to 2022.

Country	TB MR	2018	2019	2020	2021	2022	Total
Benin	Number confirmed	27	29	31	33	34	154
	Number treated	24	25	24	28	24	125
	Proportion treated (%)	88.9	86.2	77.4	84.8	70.6	81.2
Burkina Faso	Number confirmed	101	74	63	82	77	397
	Number treated	88	62	55	75	68	348
	Proportion treated (%)	87.1	83.8	87.3	91.5	88.3	87.7
Cameroon	Number confirmed	176	195	171	138	174	854
	Number treated	151	169	151	108	152	731
	Proportion treated (%)	85.8	86.7	88.3	78.3	87.4	85.6
Niger	Number confirmed	72	79	79	97	134	461
	Number treated	65	65	69	87	121	407
	Proportion treated (%)	90.3	82.3	87.3	91.7	90.3	88.4
Senegal	Number confirmed	99	58	75	79	83	394
	Number treated	86	49	65	69	81	361
	Proportion treated (%)	86.8	84.4	86.7	87.3	97.6	91.6
Togo	Number confirmed	23	20	16	18	16	93
	Number treated	16	18	12	12	14	72
	Proportion treated (%)	69.6	90.0	75.0	66.7	87.5	77.4
All countries	Number confirmed	498	455	435	447	518	2353
	Number treated	430	388	376	379	460	2033
	Proportion treated (%)	86.3	85.3	86.4	84.8	88.8	86.4

**Table 4:** Gap between the expected number of cases and the number of cases reported by country from 2018 to 2022.

Country	TB MR	2018	2019	2020	2021	2022
Benin	Expected number of cases	90	89	89	88	78
	Number of cases reported	27	29	31	33	34
	Gap in percentage (%)	70	67.4	65.2	62.5	56.4
Burkina Faso	Expected number of cases	210	200	190	190	140
	Number of cases reported	101	74	63	82	77
	Gap in percentage (%)	51.9	63.0	66.8	56.8	45.0
Cameroon	Expected number of cases	920	880	850	810	750
	Number of cases reported	176	195	171	138	174
	Gap in percentage (%)	80.9	77.8	79.9	83.0	76.8
Niger	Expected number of cases	600	620	630	650	620
	Number of cases reported	72	79	79	97	134
	Gap in percentage (%)	88	87.3	87.5	85.1	78.4
Senegal	Expected number of cases	280	270	270	270	220
	Number of cases reported	99	58	75	79	83
	Gap in percentage (%)	64.6	78.5	72.2	70.7	62.3
Togo	Expected number of cases	33	32	30	29	22
	Number of cases reported	16	18	12	11	16
	Gap in percentage (%)	51.5	43.7	60.0	62.1	27.3
All countries	Expected number of cases	2133	2091	2059	2037	1830
	Number of cases reported	491	453	431	440	518
	Gap in percentage (%)	77.0	78.3	79.1	78.4	71.7

**Table 5:** Number of confirmed XDR-TB cases put on treatment from 2018 to 2022 *.

Country	XDR-TB	2018	2019	2020	2021	2022	Total
Benin	Number confirmed	0	1	0	0	0	1
	Number treated	0	1	0	0	0	1
Burkina Faso	Number confirmed	2	4	0	3	2	11
	Number treated	2	4	0	3	2	11
Cameroon	Number confirmed	3	5	2	1	1	12
	Number treated	3	5	2	1	1	12
Niger	Number confirmed	3	1	2	11	0	17
	Number treated	3	1	2	11	0	17
Senegal	Number confirmed	0	3	4	1	2	10
	Number treated	0	3	4	1	2	10
Togo	Number confirmed	1	2	0	0	0	3
	Number treated	1	2	0	0	0	3
All countries	Number confirmed	9	16	6	16	5	52
	Number treated	9	16	6	16	5	52

* For the time period between 2018 and January 2021, we used the previous XDR definition. However, for patients put on treatment from February 2021 onwards, we used the new WHO definition provided in the Introduction.

**Table 6:** Treatment protocols by country *.

Country	Treatment Protocols
Benin	MDR/RR-TB 2013–2020: 4 KmMfxPtoHCfzEZ/5 MfxCfzEZ From August 2020: 4BdqLfxPtoHCfzEZ/2BdqLfxCfzEZ/3LfxCfzEZ pre-XDR-TB and XDR-TB 6Lzd hH BdqCfzZ/14Lzd (l-M-V) Cfz Z
Burkina Faso	MDR/RR-TB 2014–2018: 4Km-Mfx-Pto-E-Z-H_hd_ -Cfz/5 Mfx-E-Z-Cfz 2019: 4Am-Mfx-Pto-E-Z-H_hd_ -Cfz/5 Mfx-E-Z-Cfz From 2020: 4–6 Bdq[6]-Lfx[Mfx]-Pto-E-Z-H_hd_ -Cfz/5 Lfx[Mfx]-E-Z-Cfz pre-XDR-TB and XDR-TB 2018–August 2021: 6 Am-Bdq-Lfx-Lzd-Cfz-Z-Eto-PAS/14 Lfx-Cfz-Z-Eto-PAS Since August 2021: 6BPaL
Cameroon	MDR/RR-TB 2013–2018: 4–6 Km Hh Pto Mfx Cfz E Z/5 Mfx Cfz E Z 2018–2020: 4–6 Am Hh Pto Mfx Cfz E Z/5 Mfx Cfz E Z Since 01 June 2021: 4–6 Bdq (6) Hh Pto Mfx Cfz E Z/5 Mfx Cfz E Z pre-XDR-TB and XDR-TB 2013–2022: 4–6 Lzd-Hh-Bdq-Dlm-Cfz-Z/8 Bdq-Dlm-Cfz-Z
Niger	MDR/RR-TB 10/2015 to present: Lzd has replaced ISL for hearing loss (oral) From 2021: fully oral regimes in operational research conditions - TB-RR/FQ-S: 4–6 Lzd-Hh-Pto-Lfxh-Bdq-Cfz-Z/5 Lfxh-Bdq-Cfz-Z - TB-RR/FQ-R: 4–6 Lzd-Hh-Bdq-Dlm-Cfz-Z/5 Bdq-Dlm-Cfz-Z pre-XDR-TB and XDR-TB Individual plan
Senegal	MDR/RR-TB 2015 (Q3)–2019: 4–6 Km-Mfx-Pto-Cfz-H_FD_-E-Z/5 Mfx-E-Cfz-Z 6–8 Am-Lfx-Eto-PAS- Cs-Z/12–14 Z-Lfx-Eto- PAS (RR/MR TB cases not eligible for short regimen: pregnant women, extrapulmonary) 2020–2021: 4 (Am-Mfx-Pto-Cfz--E-Z)/5 (Mfx-E-Cfz-Z) Since 2021 Adults and children aged 6 and over: - short regime (4–6 Bdq (6 months) -Lfx-Cfz-Z-E-Hh-Eto/5 Lfx Cfz-Z-E) - Or long diet (6–8 Bdq (6 months) -Lfx-Lzd-Cs-Cfz-Z/12–14 (Lfx-Lzd-Cfz-Z) Children aged 5 and under (slightly modified schemes) - short regimes (4–6 Dlm-Lfx-Cfz- -Z-E-Hh-Eto/5 Lfx-Cfz-Z-E) Or long-term (6–8 Dlm-Lfx-Lzd-Cs-Cfz-Z/12–14 (Lfx-Lzd-Cfz-Z) pre-XDR-TB and XDR-TB 2020–2021: 6 Cm-Bdq-Lfx-Lzd-Cfz-Z-Eto-PAS/14 Lzd-Lfx-Cfz-Z-Eto-PAS 6 (Bdq-Lfx-Lzd-Cs-Cfz-Z/12–14 (Lfx-Lzd-Cs-Cfz-Z)
Togo	MDR/RR-TB - 2015–2021: 4 Am-Mfx-Pto-Hh-Cfz-E-Z/5 Mfx-Cfz-E-Z - October 2021: 4 Bdq-Lfx-Pto-Cfz-Z-H-E/2Bdq-Lfx-Z-E/3Lfx-Cfz-E-Z pre-XDR-TB and XDR-TB - From 2019: 6 Dlm-H-Bdq-Lzd-Cfz-Z/6Bdq-Lzd-Cfz-Z/8Lzd-Cfz-Z

* Km: Kanamycin, Pto: Prothionamide, H: Isoniazid, Cfz: Clofazimine, E: Ethambutol, Z: Pyrazinamid, Mfx: Moxifloxacin, Bdq: Bedaquiline, Lfx: Levofloxacin, Lzd: Linezolid, H_h_: high dose isoniazid, Am: Amikacin, Eto: Ethionamide, PAS: Para-aminosalicylic acid, Dlm: Delamanid, Cs: Cycloserine, Cm: Capreomycin, BPaL is a regimen containing the following drugs: Bedaquiline, Pretomanid and Linezolid.

**Table 7:** Care organization by country.

Country	Organisation of Care
Benin	- Single care centre, with decentralisation in the continuation phase for certain cases - Hospital accommodation for strict ODD throughout treatment - Nutritional and financial support to facilitate travel for TDO - Follow-up examinations free of charge
Burkina Faso	- Initiation of treatment in the country’s 2 pneumology departments (CHU) - Hospitalisation if necessary - Outpatient follow-up at the CDT of origin or at the university hospital (13 outpatient centres) - Follow-up examinations free of charge - Nutritional and financial support for TDO travel expenses - Decentralisation of DR-TB management in RHCs and HDs from 2020 onwards
Cameroon	- 11 TBMR care centres - Intensive phase hospital accommodation - Outpatient continuation phase (10 outpatient centres) - Follow-up examinations free of charge - Nutritional and financial support for TDO travel expenses
Niger	- 4 TB/MR treatment units (Niamey, Maradi, Zinder and Tahoua) - Intensive phase hospital accommodation for serious cases only - Outpatient continuation phase - Free follow-up examinations and medicines to manage adverse effects - Nutritional and financial support for TDO travel expenses - Home visits and advice for families
Senegal	- Outpatient treatment since 2010 from start to finish - Gradual decentralisation based on mapping of patients screened, 37 sites operational by April 2023 - Hospitalisation of patients with complications at the MDR-TB management reference unit of the Fann pneumology department in Dakar - Follow-up examinations free of charge - Nutritional and financial support for TDO travel expenses
Togo	- A TB/MR Care Unit (Pneumology Department, Sylvanus Olympio University Hospital, Lomé) - Intensive phase hospital accommodation - Outpatient continuation phase - Follow-up examinations free of charge - Nutritional and financial support for travel expenses to ensure the TDO

**Table 8:** Treatment outcome for MDR-TB for all schemes, 2018–2021.

Country	Treatment Results	Year			
		2018	2019	2020	2021
Benin	Number of patients (N)	27	29	31	33
	Therapeutic success n (%)	19 (70.4)	25 (86.2)	26 (83.9)	27 (81.8)
	Failure n (%)	0 (0.0)	0 (0.0)	0 (0.0)	0 (0.0)
	Loss to follow-up n (%)	3 (11.1)	0 (0.0)	1 (3.2)	2 (6.1)
	Deceased n (%)	5 (18.5)	4 (13.8)	3 (9.7)	4 (12.1)
	Not assessed n (%)	0 (0.0)	0 (0.0)	1 (3.2)	0 (0.0)
Burkina Faso	Number of patients (N)	101	74	63	82
	Therapeutic success n (%)	63 (62.4)	43 (58.1)	41 (65.1)	44 (53.7)
	Failure n (%)	3 (3.0)	3 (4.1)	1 (1.6)	5 (6.1)
	Loss to follow-up n (%)	5 (5.0)	14 (18.9)	4 (6.3)	8 (9.8)
	Deceased n (%)	30 (29.7)	14 (18.9)	17 (27.0)	25 (30.5)
	Not assessed n (%)	0 (0.0)	0 (0.0)	0 (0.0)	0 (0.0)
Cameroon	Number of patients (N)	176	195	171	138
	Therapeutic success n (%)	117 (66.5)	146 (74.9)	76 (44.4)	NAv *
	Failure n (%)	3 (1.7)	3 (1.5)	5 (2.9)	NAv
	Loss to follow-up n (%)	8 (4.5)	6 (3.1)	4 (2.3)	NAv
	Deceased n (%)	16 (9.1)	15 (7.7)	16 (9.4)	NAv
	Not assessed n (%)	32 (18.2)	25 (12.8)	70 (40.9)	NAv
Niger	Number of patients (N)	72	79	79	87
	Therapeutic success n (%)	55 (76.4)	68 (86.1)	59 (74.7)	67 (76.7)
	Failure n (%)	4 (5.5)	1 (1.2)	3 (3.8)	1 (1.7)
	Loss to follow-up n (%)	1 (1.4)	4 (5.1)	3 (3.8)	7 (8.3)
	Deceased n (%)	12 (16.7)	6 (7.6)	14 (17.7)	12 (13.3)
	Not assessed n (%)	0 (0.0)	0 (0.0)	0 (0.0)	0 (0.0)
Senegal	Number of patients (N)	86	49	65	69
	Therapeutic success n (%)	75 (87.2)	40 (81.6)	54 (83.0)	NAv
	Failure n (%)	3 (3.4)	3 (6.1)	2 (3.0)	NAv
	Loss to follow-up n (%)	2 (0.33)	1 (2)	1 (1.5)	NAv
	Deceased n (%)	6 (6.9)	5 (10.2)	8 (12.3)	NAv
	Not assessed n (%)	0	0	0	NAv
Togo	Number of patients (N)	16	18	12	11
	Therapeutic success n (%)	13 (81.3)	14 (77.8)	8 (66.7)	10 (90.9)
	Failure n (%)	1 (6.3)	0 (0.0)	0 (0.0)	0 (0.0)
	Loss to follow-up n (%)	0 (0.0)	0 (0.0)	0 (0.0)	0 (0.0)
	Deceased n (%)	2 (12.5)	4 (22.2)	4 (33.3)	1 (9.1)
	Not assessed n (%)	0 (0.0)	0 (0.0)	0 (0.0)	0 (0.0)
All countries	Number of patients (N)	478	444	421	420
	Therapeutic success n (%)	342 (71.5)	336 (75.7)	264 (62.7)	NAp **
	Failure n (%)	14 (2.9)	10 (2.3)	11 (2.6)	NAp
	Loss to follow-up n (%)	19 (4.0)	25 (5.6)	13 (3.1)	NAp
	Deceased n (%)	71 (14.9)	48 (10.8)	62 (14.7)	NAp
	Not assessed n (%)	32 (6.7)	25 (5.6)	71 (16.9)	NA

* NAv: not available; ** NAp: not applicable.

**Table 9:** Outcome of TB-XDR treatment from 2018 to 2021.

Country	Treatment Results	Year			
		2018	2019	2020	2021
Benin	Number of patients (N)	0	1	0	0
	Therapeutic success n (%)	0	1	0	0
	Failure n (%)	0	0	0	0
	Loss to follow-up n (%)	0	0	0	0
	Deceased n (%)	0	0	0	0
	Not assessed n (%)	0	0	0	0
Burkina Faso	Number of patients (N)	2	4	0	3
	Therapeutic success n (%)	1	1	0	1
	Failure n (%)	0	0	0	0
	Loss to follow-up n (%)	0	0	0	0
	Deceased n (%)	1	2	0	2
	Not assessed n (%)	0	1	0	0
Cameroon	Number of patients (N)	3	5	2	1
	Therapeutic success n (%)	1	2	1	1
	Failure n (%)	2	0	0	0
	Loss to follow-up n (%)	0	0	1	0
	Deceased n (%)	0	3	0	0
	Not assessed n (%)	0	0	0	0
Niger	Number of patients (N)	6	4	4	11
	Therapeutic success n (%)	4	4	4	9
	Failure n (%)	0	0	0	0
	Loss to follow-up n (%)	0	0	0	0
	Deceased n (%)	2	0	0	2
	Not assessed n (%)	0	0	0	0
Senegal	Number of patients (N)	0	3	4	1
	Therapeutic success n (%)	0	1	2	0
	Failure n (%)	0	1	0	0
	Loss to follow-up n (%)	0	0	0	0
	Deceased n (%)	0	1	2	1
	Not assessed n (%)	0	0	0	0
Togo	Number of patients (N)	1	2	0	0
	Therapeutic success n (%)	0	2	0	0
	Failure n (%)	0	0	0	0
	Loss to follow-up n (%)	0	0	0	0
	Deceased n (%)	1	0	0	0
	Not assessed n (%)	0	0	0	0
All countries	Number of patients (N)	12	19	10	16
	Therapeutic success n (%)	6	11	7	11
	Failure n (%)	2	1	0	0
	Loss to follow-up n (%)	0	0	1	0
	Deceased n (%)	4	6	2	5
	Not assessed n (%)	0	1	0	0

**Table 10:** Tools used in the countries.

Country	Tools
Benin	- MDR-TB treatment register - MDR-TB patient file - Treatment result form for DR-TB patients - Laboratory records - MDR-TB case report form - Xpert MTB/Rif monthly report form - Interconnecting Data To Care—ABLAB
Burkina Faso	- MDR-TB treatment register - MDR-TB patient file - Treatment result form for DR-TB patients - Laboratory records - Contact investigation form - Form for reporting cases put on MDR-TB treatment - Xpert MTB/Rif monthly report form - TB tracker implementation
Cameroon	- MDR-TB treatment register - MDR-TB patient file - Treatment result form for DR-TB patients - Laboratory records - Form for reporting cases put on MDR-TB treatment - Xpert MTB/Rif monthly report form - Data To Care system
Niger	- Patient File - DHIS2 input form - Online and paper registers (laboratory and care) - Registers for LPA and for cultivation at LNR - Quarterly reports from TB/MR units and Xperts sites - Adsm reports
Senegal	- GeneXpert Registry - LPA register - Crop register - TB treatment register MR - Patient file TB MR - TB MR quarterly report forms - Setting up reports in the DHIS2 platform - Registration and processing folder settings in the TB tracker
Togo	- MDR tuberculosis treatment registry - Multidrug-resistant tuberculosis electronic screening registry - MDR-TB patient file - MDR-TB patient’s hospitalization notebook - TB tracker implementation - Implementing DataToCare (CETA)

## Data Availability

The data that support the findings of the study are available from the National Tuberculosis Programme Department of each country upon reasonable request.
